# Case Report: Review of CT Findings and Histopathological Characteristics of Primary Liver Carcinosarcoma

**DOI:** 10.3389/fgene.2021.638636

**Published:** 2021-06-17

**Authors:** Lu Huang, Lijian Lu

**Affiliations:** ^1^Department of Infectious Diseases, The First Affiliated Hospital of Guangxi Medical University, Nanning, China; ^2^Department of Radiology, The Wuming Affiliated Hospital of Guangxi Medical University, Nanning, China

**Keywords:** liver, tumora, tomography, x-ray computed, diagnosis

## Abstract

**Objectives:** The aim of the present study was to describe the computed tomography (CT) characteristics of primary liver carcinosarcoma (PLCS) and to explore the pathological basis for the diagnosis of primary hepatocellular carcinoma sarcoma.

**Methods:** Three male patients with PLCS were included in the present retrospective research, and the age was ranged from 52 to 63 years. The plain CT scan and third-stage enhancement scan were performed on patients. The pathological characteristics were analyzed. Stomachache was the main clinical symptoms of the three patients. Cirrhosis background was confirmed in one patients, and chronic Hepatitis B background was confirmed in other two patients.

**Results:** According to the results of CT, the inner diameter of the tumors ranged from 8.6 to 27.0 cm. The fibrous pseudocapsule around the tumor tissues was observed in two patients. Tumor tissues from all three patients were composed of sarcomatous and carcinomatous components. For carcinomatous components, hepatocellular carcinoma was observed in one patient and cholangiocarcinoma was observed in the other two patients. For sarcomatous components, angiosarcoma was observed in two patients and malignant fibrous histiocytoma was observed in another one patient. The tumor tissues were visualized as heterogeneous low density with large sheets of necrotic cystic lesions or thick-walled areas of multilocular cystic lesions using the plain CT scan. Edge-to-center filling and strengthening lesions, mild to moderate enhanced parenchyma at the arterial phase, and isodensity between the tumor parenchyma and the surrounding liver parenchyma at the portal vein phase or delayed phase were observed using the third-stage enhancement scan.

**Conclusions:** CT characteristics observed in the present study were of great benefit for the diagnosis of PLCS.

## Introduction

Primary liver carcinosarcoma (PLCS) is defined as a malignant tumor concomitantly composed of a mixture of sarcomatous and carcinomatous by the World Health Organization (WHO), which is either hepatocyte-derived or cholangiocyte-derived or mixed. Currently, the pathological mechanism underlying PLCS is unclear (Xiang et al., 2015). PLCS is a type of rare and complex hepatic malignant tumor with aggressive growth characteristics, propensity for recurrence, and a poor prognosis (Li et al., 2016). Preoperative diagnosis of PLCS is typically challenging, which relies on the postoperative pathological examination (Shu et al., 2010). Both epithelial and mesenchymal sarcoma components can be observed on PLCS tumor tissues using a microscope, and immunohistochemical assay plays an important role for the further diagnosis of PLCS (Lao et al., 2007). In the present case-series report, three patients diagnosed with PLCS using surgical pathology in our hospital were included. The purpose of the present study was to describe the clinical, histopathological, and imaging characteristics of PLCS and to document the associated imaging presentations and results.

## Materials and Methods

The present retrospective research was authorized by the institutional research ethics committee of The First Affiliated Hospital of Guangxi Medical University. Informed consent was not applicable. The image data from all the three patients diagnosed with PLCS from January 2011 and February 2018 were analyzed. Two pathologists confirmed the pathological diagnosis of the cases. The medical records were consulted to determine the clinical manifestation, treatment, and outcome of the cases.

Three patients underwent the plain CT scan and third-stage enhancement scan (64 MDCT TK LIGHT SPEED GE Medical System). The scanning parameters were shown as the following: slice thickness: 5 mm; pitch: 1.375; bed speed: 5.5 mm/s; tube voltage: 120 kV; and tube current: 100 mA. Multi-planar recombination (MPR) was used for post-processing of images. Enhanced scanning was performed using a high-pressure syringe. The contrast agent administered was iopromide (includes 300 mg/mL of iodine) for a total of 70–85 mL with a flow rate of 3 mL/s.

Imaging results were reviewed independently by two abdominal imaging radiologists with 15 and 16 years of working experience, which were cross-checked by another radiologist to obtain the consistent conclusion. In the present study, the characteristics of the results of CT scans on tumors were evaluated, including position, size, relationship with hepatic envelope, edge, uniformity of density, and presence of adipose tissue, hemorrhage, cystic components, calcification, and vascular tumor.

## Results

### Clinical Characteristics

Three male patients (52–63 years old) with PLCS who were treated at our hospital between January 2011 and February 2018 were included in the present study. All three patients were admitted to the hospital due to abdominal pain and a space-occupying lesion in the liver tissues. Two patients had a history of chronic hepatitis B, and one patient had a history of cirrhosis. A significant elevated level of carcinoembryonic antigen 199 (CA199) was observed in two patients, and an elevated level of alpha-fetoprotein (AFP) was observed in another one patients. All three patients had normal levels of carcinoembryonic antigen (CEA) (Table 1).

**Table 1:** Clinical features of three patients with PLCS.

**Case**	**age (years)**	**Sex**	**Main clinical symptoms**	**Liver disease**	**CA125**	**CA199**	**CEA**	**AFP**	**Prothrombin**
1	52	male	Upper abdominal pain	Chronic hepatitis B	-	-	-	-	-
2	42	male	Right upper quadrant pain	Cirrhosis	-	+	-	+	+
3	63	male	Right upper quadrant pain	Chronic hepatitis B	-	+	-	-	-

### Pathological Characteristics

Two experienced abdominal pathologists individually analyzed the pathological data, which were cross-checked by another experienced abdominal pathologist. The maximum diameter of the lesion was ranged from 8.6 to 27.0 cm. The tangent plane of the lesions from all three patients was grayish white. A pseudo-envelope of fibrous tissue around the tumor was observed in two of the patients.

### PLCS Consisted of Cancerous and Sarcoma Components

**Figure 1:**
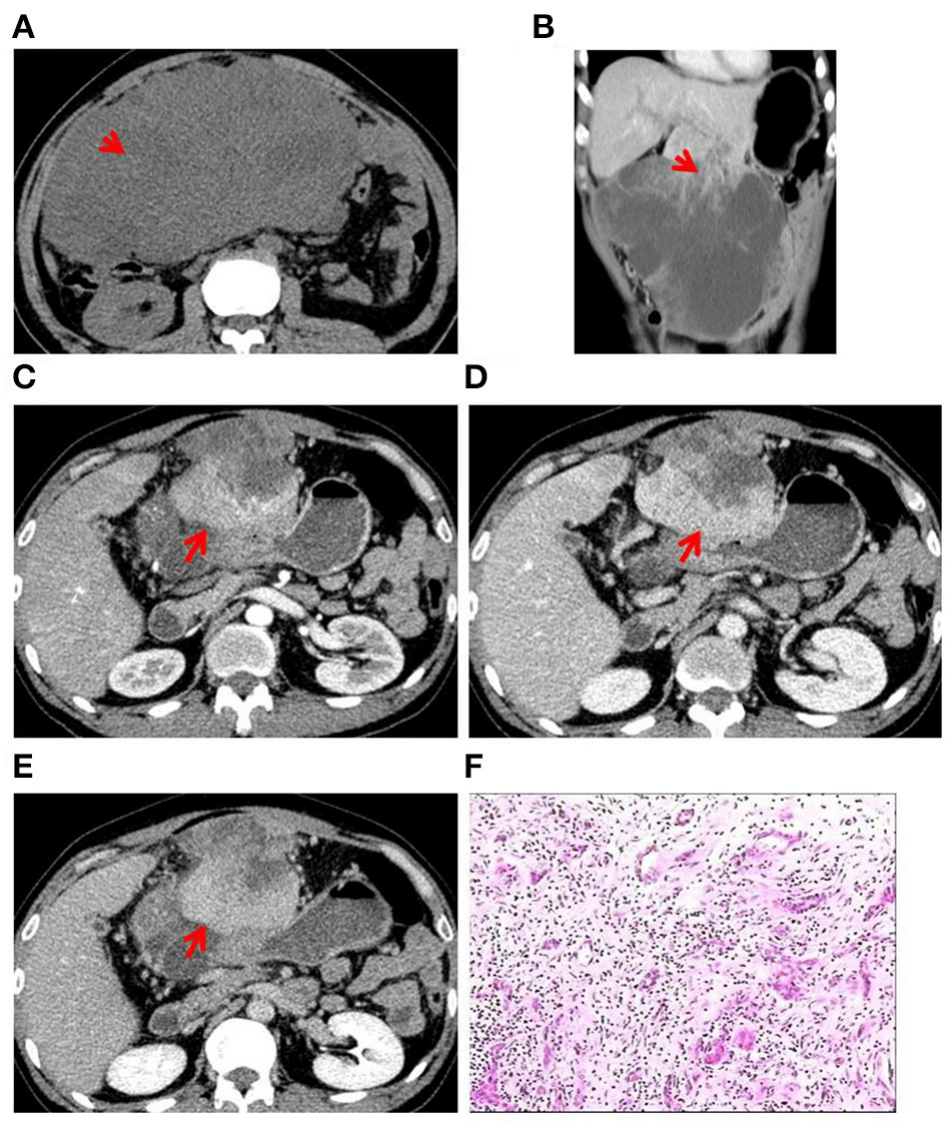
Case 1: A 52-year-old man presented with upper abdominal pain since 3 months. **(A)** CT scan showed heterogeneously hypo-dense lesions (red arrow). **(B)** A large exogenous mass in the S3 segment of the liver was observed with irregular thick-walled areas inside the lesion (red arrow). **(C)** Contrast-enhanced CT in the arterial phase showed uneven mild to moderate enhancement of the lesion with intratumoral vessels and that the tumor has invaded the anterior abdominal wall (red arrow). **(D)** CT scan in the portal-vein phase showed gradual filling of the lesion from the edge to the center (red arrow). **(E)** CT scan in the prolongation phase showed gradual filling of the lesion from the edge to the center (red arrow). **(F)** Histopathological examination of surgical specimen: the carcinomatous component (cholangiocarcinoma) is interspersed with the sarcomatous component (angiosarcoma).

**Figure 2:**
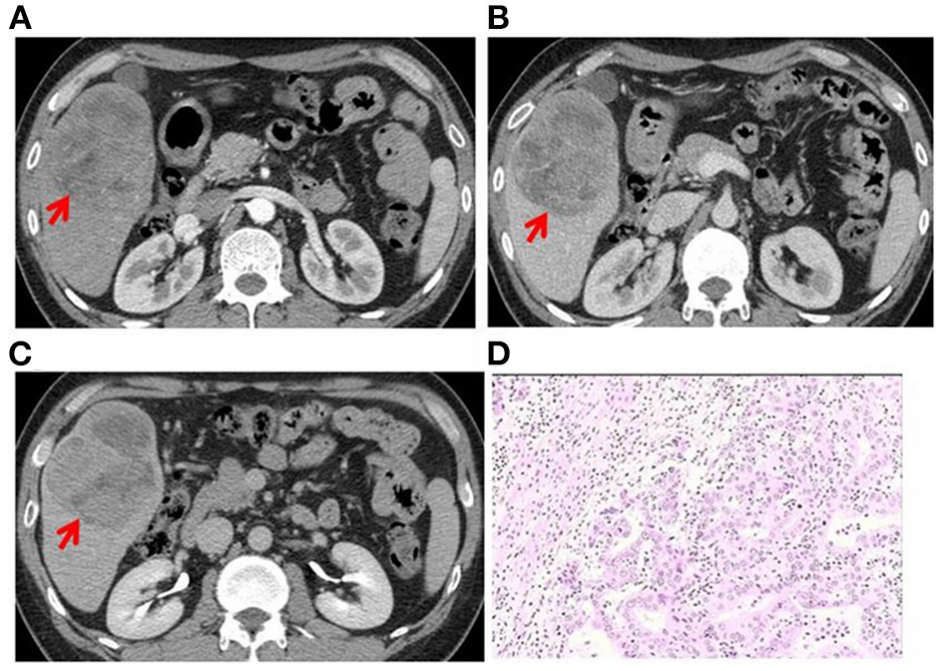
Case 2: A 42-year-old man had abdominal pain in the right upper quadrant since 1 month. **(A)** A round mass in the S5 segment of the liver was observed. CT scan in the arterial phase showed uneven, mild to moderate enhancement (red arrow). **(B)** CT scan in the portal-vein phase showed gradual filling of the lesion from the edge to the center, with irregular thick-walled sac-variable regions (red arrow). **(C)** CT scan in the prolongation phase showed gradual filling of the lesion from the edge to the center, with irregular thick-walled sac-variable regions (red arrow). The tumor was surrounded by a pseudocapsule. **(D)** Histopathological examination of the surgical specimen shows PLCS. The carcinomatous component (cholangiocarcinoma) was interspersed with the sarcomatous component (angiosarcoma).

**Figure 3:**
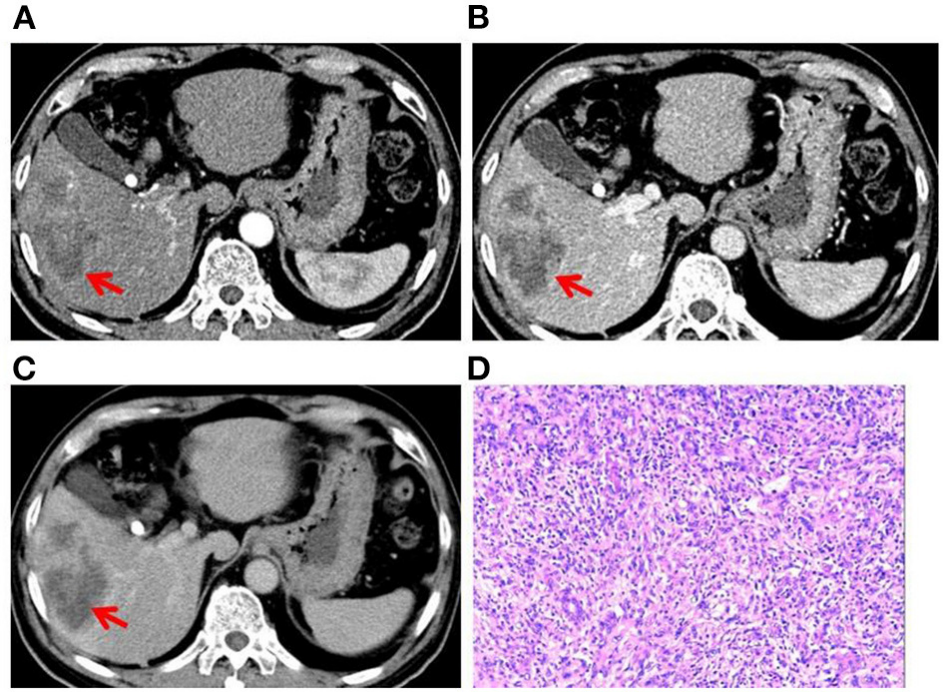
Case 3: A 63-year-old man presented with pain in the right upper quadrant since 1 month. **(A)** A round mass in the S5 segment of the liver. CT scan in the arterial phase showed uneven, mild to moderate enhancement of the lesion. Tumor vessels were visible in the tumor. Irregular thick-walled sac-variable regions (red arrows) were seen in the lesion. **(B)** CT scan in the portal-vein phase showed gradual filling of the lesion from the edge to the center, with irregular thick-walled sac-variable regions (red arrow). **(C)** CT scan in the prolongation phase showed gradual filling of the lesion from the edge to the center, with irregular thick-walled sac-variable regions (red arrow). The tumor was surrounded by a pseudocapsule. **(D)** Histopathological examination of surgical specimen showing PLCS. Cancerous tissue (cholangiocarcinoma) was interspersed with sarcomatous tissue (malignant fibrous tissue).

Tumor tissues composed of both cancerous and sarcomatous components interspersed with each other were observed in all three patients. For carcinomatous components, hepatocellular carcinoma was observed in one patient and cholangiocarcinoma was observed in the other two patients. For sarcomatous components, angiosarcoma was observed in two patients and malignant fibrous histiocytoma was observed in another one patient Figures 1F, 2D, 3D. Immunohistochemical results were shown as follows: Hep-1 (+) (one patient), AFP (+) (one patient), CK (+) (one patient), CK19 (+) (two patients), Vim (+) (two patients), CD34 (+) (two patients), and CD68 (+) (two patient), which were consistent with the diagnosis of PLCS Table 2.

**Table 2:** Pathological features of three cases with PLCS.

**Item**	**Case 1**	**Case 2**	**Case 3**
Pathology	One large liver mass, inner diameter: 27 cm, grayish cut surface, large necrotic area, and old bleeding	One large liver mass, inner diameter: 15 cm, grayish cut surface, some areas accompanied by hemorrhage and necrosis, and fibrous tissue wrapping around the tumor	One large liver mass, inner diameter: 8.6 cm, grayish cut surface, large necrotic area and old bleeding, and fibrous tissue wrapping around the tumor
Microscopy	The cancer tissue and the sarcoma tissue arranged in a mixed manner; cholangiocarcinoma in the cancer tissue, and angiosarcoma in the sarcoma. Immunohistochemistry CK19, Vim, CD34 (+)	The cancer tissue and the sarcoma tissue arranged in a mixed manner; hepatocellular carcinoma in the cancer tissue and angiosarcoma in the sarcoma. The sarcoma is an angiosarcoma, and the fibrous tissue is surrounded around the tumor. Immunohistochemistry AFP, Hep-, CK, CD34(+)	The cancer tissue and the sarcoma tissue arranged in a mixed manner; cholangiocarcinoma in the cancer tissue; malignant fibrous tissue tumor in the sarcoma; Immunohistochemistry CK19,CD68/34, Vim(+)

### CT Imaging Findings

Two experienced abdominal radiologists independently analyzed the imaging data, which were cross-checked by another experienced abdominal pathologist. All three patients had a single lesion in the liver, and CT scan showed an uneven and low-density zone (Figure 1A). An irregular and exogenous shaped tumor lesion was found to be located in the left lobe of the liver of case 1 (Figure 1B), while a pseudo-envelope with a clear boundary was formed around the tumor lesion in case 2 and case 2 (Figures 2A,B, 3A,B). The tumor boundary was blurred, and there was no pseudo-envelope formation in case 1. Moreover, all three lesions showed mixed density and irregular thick-walled separation changes in the cystic zone (Figures 1B, 2A–C, 3A–C). No sign of calcification or intratumoral bleeding was observed in any of the patients.

According to the results of enhanced CT scan, the tumor margins in lesions from all patients were gradually filled and intensified toward the center (Figures 1C–E, 2A–C, 3A–C). Uneven and mildly enhanced tumor parenchyma and enriched tortuous tumor vessels were observed in the arterial phase (Figures 1C, 2A, 3A). Isodensity with hepatic parenchyma was observed in the portal vein and lag phase (Figures 1D,E, 2B,C, 3B,C). Invasion into the left branch of the portal vein and established tumor thrombus were observed in case 1, in which one lesion broke through the hepatic liver capsule into adjacent tissues. Tumor recurrence and distant metastasis were observed in case 2 and case 2 within 3 months after operation (Table 3).

**Table 3:** CT features of three cases with PLCS.

**Item**	**Location**	**Number of lesions**	**Morphology**	**Edge**	**Density**	**Calcification**	**Hemorrhage**	**Thick-walled sac change**	**Enhanced features**	**Pseudo capsule**	**Vascular invasion**	**Invasion of adjacent tissue**	**Bile duct invasion**	**Ascites**	**Metastasis**	**Recurrence**
Case 1	S3/external	Single	Irregular	Blurry	Uneven	-	-	+	The edge is gradually filled and enhanced toward the center, and iso-density area is visible in the portal-vein or delayed phase.	-	+	+	-	-	Left abdominal mass/2 months	2 months
Case 2	S5	Single	Round	Clear	Uneven	-	-	+	The edge is gradually filled and enhanced toward the center, and iso-density area is visible in the portal-vein or delayed phase.	+	-	-	-	-	Pancreatic head mass/11 months	-
Case 3	S5	Single	Irregular	Clear	Uneven	-	-	+	The edge is gradually filled and enhanced toward the center, and iso-density area is visible in the portal-vein or delayed phase.	+	-	-	-	-	-	2 months

## Discussion

In 1989, Craig et al. proposed the definition of PLCS, which refers to primary liver malignant tumor containing both hepatocellular carcinoma and sarcoma. Subsequently, PLCS is further defined by the World Health Organization as a complex malignant liver tumor composed of a mixture of hepatocellular carcinoma or cholangiocarcinoma components and sarcoma components (Seifert et al., 1990). PLCS is a rare malignant tumor with rare reports (Celikbilek et al., 2011; Liu et al., 2012; Yamamoto et al., 2014; Xiang et al., 2015; Yu, 2015; Li et al., 2018a), and the specific clinical symptoms of PLCS are uncertain. Abdominal pain and abdominal distension are regarded as the main complaints of PLCS. Approximately 80% of PLCS patients possess a history of chronic liver disease, and a significantly elevated serum alpha fetoprotein (AFP) level is observed in about 27.6% PLCS patients (Li et al., 2018a,b). In the present study, all three patients were middle-aged men with a history of chronic liver disease, which suggests that middle-aged men and chronic liver disease might be risk factors for PLCS. Among the three patients, the serum CA199 level was increased in two patients, while the serum AFP level was increased in one patient. All three patients were CEA-negative, and abnormal prothrombin level was found in one patient. These observations might be associated with the number and type of tumor cell components, which was similar to those previously reported (Li et al., 2018b). Lung and lymph nodes, peritoneum, gallbladder, omentum, stomach, diaphragm, and adrenal gland are common metastatic positions. These clinical features indicate that PLCS has high levels of aggression and is metastatic (Celikbilek et al., 2011; Yasutake et al., 2014; Gu et al., 2015; Xiang et al., 2015).

The pathogenesis of PLCS is unclear. Current evidence (Lao et al., 2007; Celikbilek et al., 2011; Yasutake et al., 2014; Gu et al., 2015) supports the theory that carcinosarcoma is monoclonal in origin. In previous studies, most PLCSs were developed in normal livers with no cirrhosis background, which indicated that tumors develop from pluripotent liver progenitor cells or stem cells. The imaging characteristics of PLCS are currently unclear due to its low incidence, which makes it difficult for radiologists to make accurate preoperative imaging diagnosis. In the present study, all three patients were misdiagnosed preoperatively as hepatocellular carcinoma. The PLCS tumor was huge, irregularly shaped, and with unclear boundaries, which was consistent with the reports described previously (Lin et al., 2013; Gu et al., 2015; Xiang et al., 2015).

Computed tomography (CT) is the most commonly used imaging method for PLCS. However, currently few reports have described the CT findings of PLCS. Previous reports have described liver cancer sarcoma as generally large and irregular low-density masses, which tends to grow across the liver segment. The boundary of tumor is blurred, and the tumor directly invades into the surrounding tissues. Necrotic cystic degeneration is commonly observed in the central part of PLCS tumor tissues. Mild to moderate intensity is reported on PLCS using enhanced CT scan (Celikbilek et al., 2011; Liu et al., 2012; Xiang et al., 2015). In the present study, the size of PLCS tumor in all three patients was relatively large, which was irregular in one patient and nearly round in the other two patients. In one patient, the tumor had broken through the liver capsule and invaded into the surrounding tissues, which were supposed to be related to the high degree of malignancy and rapid growth of liver cancer sarcoma. These observations were consistent with previous reports, in which the pseudocapsule was rarely formed in hepatocarcinoma sarcoma (Celikbilek et al., 2011; Liu et al., 2012; Xiang et al., 2015; Li et al., 2018a,b). However, in the present study, the fibrous pseudocapsule was found in two patients, which might be related to massive proliferation of liver parenchymal fibrous tissue around the tumor induced by chronic liver diseases. In all three cases, irregular thick-walled multi-segmental cystic changes were observed, which might be related to the degree of necrosis in the lesion. Moreover, in all three cases, the tumors were gradually filled and enhanced from the margin to the center in the third-stage enhancement scan. Unevenness and mild-to-moderate enhancement were observed in the arterial phase, with several distorted tumor vessels. The parenchyma density of PLCS tumor was slightly higher than that of the adjacent liver parenchyma. The parenchymal enhancement in the portal vein or delayed phase showed an equal density change. These CT imaging characteristics have not been reported in previous literature (Celikbilek et al., 2011; Liu et al., 2012; Lin et al., 2013; Yamamoto et al., 2014; Xiang et al., 2015; Li et al., 2018a,b), which indicated that the isodense area in the portal vein or delayed phase of the tumor might be related to the abundant fibrous components or vascular components in the tumor parenchyma.

As described previously, calcification and bone tissue are observed in some tumors that contain the components of chondrosarcoma and osteosarcoma, which are suggested to be important CT signs for the diagnosis of PLCS (Lai et al., 2011). However, no signs of calcification or bone tissue were observed in any of the three patients in the present study, as chondrosarcoma and osteosarcoma were not included in the sarcomatous components of the tumors. In addition, tumor recurrence and distant metastasis were observed in two patients, indicating a poor prognosis of patients with PLCS.

Currently, the diagnosis of PLCS mainly depends on pathological results. As it is difficult to distinguish PLCS with other liver malignancies, such as hepatocellular carcinoma and cholangiocarcinoma, the imaging diagnosis for PLCS is difficult. Hepatocellular carcinoma is the most common primary malignancy of the liver, which is generally derived from chronic liver disease and commonly diagnosed in the elderly population (McEvoy et al., 2013). In the CT images of hepatocellular carcinoma, a low-density mass, varying in size, and significant enhancement are regularly presented, accompanied by satellite lesions and portal vein thrombosis. Capsules on the margin were commonly observed in well-differentiated hepatocellular carcinoma. Cholangiocarcinoma occurs in the bile duct epithelium and is usually located in the left hepatic lobe. Cholangiocarcinoma is found mostly in older men with a cirrhosis background. Typical imaging features of cholangiocarcinoma include more homogeneous low-density lesions, irregular appearance, gradual centripetal enhancement, contraction of adjacent hepatic envelope, and peripheral bile duct dilatation (Lewis et al., 2010). Compared to PLCS, less extensive necrosis, cystic degeneration, or isodensity changes were observed in hepatocellular carcinoma and cholangiocarcinoma.

### Shortcomings of the Present Study

The number of cases included in the present retrospective analysis is small. The results obtained in the present study need to be further verified by more cases. In the present study, based on data collected from 2011 to 2018, the conditions of tumor recurrence and distant metastasis of patients were recorded. However, how tumor CT characteristics evolved over time was not explored yet, which will be explored in more cases in our future work.

## Conclusions

Specific CT characteristics, such as huge tumor size, large-scale cystic and necrotizing degeneration, edge-to-center filling enhancement in the enhanced CT scan, and isodensity between the tumor parenchyma and the surrounding liver parenchyma at the portal vein phase or delayed phase, may help to distinguish PLCS from other malignancies. PLCS needs to be treated by surgical resection and careful CT follow-up due to their invasiveness and poor prognosis.

## Data Availability Statement

The original contributions presented in the study are included in the article/supplementary material, further inquiries can be directed to the corresponding author.

## Ethics Statement

The studies involving human participants were reviewed and approved by Medical Ethics Committee of Wuming Hospital Affiliated to Guangxi Medical University of china. The patients/participants provided their written informed consent to participate in this study. Written informed consent was obtained from the individual(s) for the publication of any potentially identifiable images or data included in this article. All data published here are under the consent for publication.

## Author Contributions

LL designed/performed most of the investigation and data analysis. LH wrote the manuscript and provided clinical assistance. Both of the authors have read and approved the manuscript.

## Conflict of Interest

The authors declare that the research was conducted in the absence of any commercial or financial relationships that could be construed as a potential conflict of interest.
